# Decreased Expression of the GATA3 Gene Is Associated with Poor Prognosis in Primary Gastric Adenocarcinoma

**DOI:** 10.1371/journal.pone.0087195

**Published:** 2014-02-04

**Authors:** Rajiv Prasad Keshari, Wei Wang, Yu Zhang, Dan-dan Wang, Yuan-fang Li, Shu-qiang Yuan, Hai-bo Qiu, Chun-yu Huang, Yong-ming Chen, Jian-chuan Xia, Zhi-wei Zhou

**Affiliations:** 1 State Key Laboratory of Oncology in South China, Sun Yat-sen University Cancer Center, Guangzhou, China; 2 Department of Gastric and Pancreatic Surgery, Sun Yat-sen University Cancer Center, Guangzhou, China; 3 Department of Pathology, Sun Yat-sen University Cancer Center, Guangzhou, China; 4 Collaborative Innovation Center for Cancer Medicine, Guangzhou, China; IRCCS National Cancer Institute, Italy

## Abstract

**Background:**

GATA binding protein 3 (GATA3) was recently proposed to function as a tumor suppressor gene in some types of human cancer. This study aims to investigate GATA3 expression and its prognostic significance in primary gastric adenocarcinoma.

**Methodology/Principal Findings:**

Using real-time quantitative PCR (RT-qPCR) and immunohistochemical staining methods, GATA3 expression was analyzed in tissue samples from a consecutive series of 402 gastric adenocarcinoma patients who underwent resections between 2003 and 2006. The relationship between GATA3 expression, clinicopathological factors, and patient survival was investigated. The expression status of GATA3 was shown to be clearly reduced in the tumor tissue samples compared with that in the matched adjacent non-tumor tissue samples by RT-qPCR (P = 0.0014). Immunohistochemistry analysis indicated that GATA3 expression was significantly decreased in 225 of the 402 (56%) gastric adenocarcinoma cases. Reduced GATA3 expression was also observed in patients with large tumors (P = 0.017), signet ring cell carcinoma or mucinous carcinoma (P = 0.005) and tumors with lymphatic or venous invasion (P = 0.040). Additionally, reduced expression of GATA3 was more commonly observed in tumors that were staged as T4a/b (P<0.001), N3 (P<0.001), or M1 (P<0.001). Kaplan-Meier survival curves revealed that reduced expression of GATA3 was associated with poor prognosis in gastric adenocarcinoma patients (P<0.001). Multivariate Cox analysis identified GATA3 expression as an independent prognostic factor for overall survival (HR = 5.375, 95% CI = 3.647–7.921, P<0.001). To investigate the predictive ability of the models with and without containing GATA3 gene expression, Harrell's c-index was calculated as a measure of predictive accuracy of survival outcome. The c-index values revealed that model containing GATA3 expression (c-index = 0.897) had superior discrimination ability to the model without containg it (c-index = 0.811).

**Conclusions/Significance:**

Our data suggest that GATA3 plays an important role in tumor progression and that reduced GATA3 expression independently predicts an unfavorable prognosis in primary gastric adenocarcinoma patients.

## Introduction

Gastric cancer is the fourth most common type of malignant tumor worldwide and the second most common cause of cancer-related deaths each year [1]. An estimated number of one million new cases arise per year [1]. The treatment consists of a combination of surgery, chemotherapy, and radiation therapy. However, nearly 60% of patients succumb to gastric cancer, even after curative resection or adjuvant therapy [2]. The outcome of patients is difficult to predict with classical histological classifications because gastric cancer is a heterogenous disease with respect to both histology and genetics. Tumor progression is considered to be a multifactorial and multistep process that involves the activation of oncogenes and the inactivation of tumor suppressor genes at different stages. The confirmation of several new oncogenes and tumor suppressor genes that are associated with gastric cancer may be useful for early diagnosis and the development of molecularly targeted therapies [3], [4]. To improve the prognosis of gastric adenocarcinoma, a better understanding of the molecular mechanisms of cancer progression and the development of new therapeutic tools based on these mechanisms are required [3], [5]–[7].

Gastrointestinal morphogenesis results from a delicately controlled interplay of cell interactions, epithelial mesenchymal crosstalk, and the complex regulatory network of signaling peptides and transcription factors. The regulatory proteins that are important in prenatal development continue to have vital roles in the mature gastrointestinal tract in maintaining stem cell populations, determining cell fate, programming differentiation, and coordinating tissue renewal [8]. Growing evidence suggests that the GATA transcription factors play crucial roles in gastrointestinal morphogenesis and the maintenance of the normal epithelium of the mature alimentary tract [8].

GATA binding protein 3 (GATA3) is 1 of 6 members of the GATA zinc finger transcription factor family. It binds to the DNA sequence [A/T]GATA[A/G] and plays an important role in promoting and directing cell proliferation, development, and differentiation in many tissues and cell types [9], [10], including the luminal glandular epithelial cells of the mammary gland [11]–[14], T lymphocytes [15], [16], thymocytes [17], [18], adipose tissue [19], the kidney [20], the sympathetic nervous system [21], and the hair follicles of the skin [22]. Together with S100P, it has recently been reported to be a useful immunohistochemical marker for the detection of urothelial carcinoma and ovarian Brenner tumors [23]–[25]. GATA3 overexpression has been observed in breast carcinomas by complementary DNA microarray analysis [26]. Low GATA3 expression has also been suggested to correlate with poor prognosis in breast cancer [27], [28].

However, to the best of our knowledge, no previous reports exist concerning the expression status of GATA3 and the prognostic value of this protein in primary gastric adenocarcinoma. Therefore, in this study, the expression of GATA3 in primary gastric adenocarcinoma was evaluated using real-time quantitative PCR (RT-qPCR) and immunohistochemistry. Additionally, we identified the relationship between GATA3 expression and clinicopathological features, and we evaluated the prognostic value of the GATA3 expression level to post-resection survival in gastric cancer.

## Results

### Analysis of GATA3 mRNA Expression by RT-qPCR

**Figure 1:**
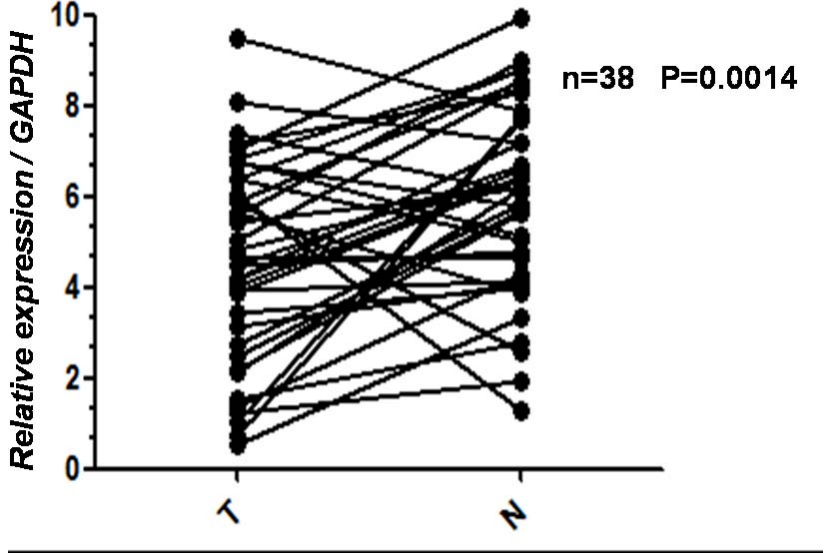
Decreased mRNA expression of GATA3 in gastric cancer tissues as assessed by real time quantitative PCR (n = 38, P = 0.0014). T = Tumor, N = Normal.

The levels of GATA3 mRNA in 38 pairs of resected specimens (tumor tissue samples and matched adjacent non-tumor tissue samples) from eligible gastric cancer patients were estimated by RT-qPCR. The GATA3 mRNA levels were significantly reduced in 28 (74%) of the tumor tissue samples compared with the adjacent non-tumor tissue samples (P = 0.0014, Figure 1).

### Immunohistochemical Staining of GATA3

**Figure 2:**
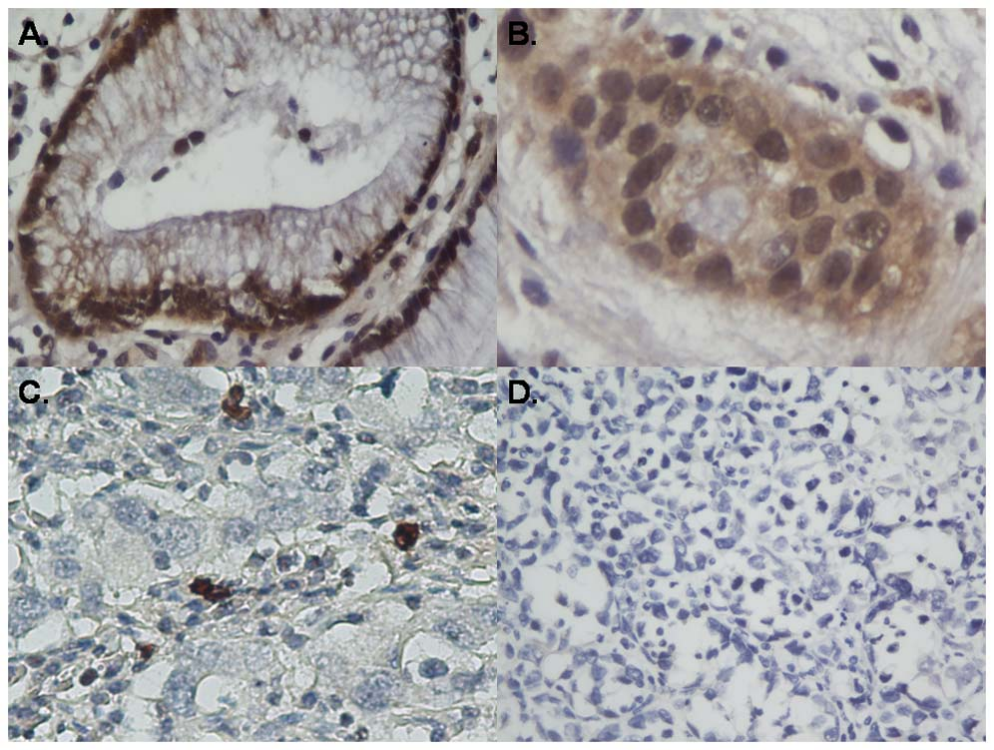
GATA3 protein expression in gastric cancer surgical specimens shown by immunohistochemistry. (A) Strong GATA3 staining (+++) was observed in noncancerous gastric mucosa. (B) GATA3 staining (++) in well differentiated gastric cancer. (C) GATA3 staining (+) in poorly differentiated gastric cancer. (D). Negative GATA3 staining (−) in mucinous carcinoma.

To obtain further insight into the effect and prognostic value of GATA3 expression in gastric cancer patients, paraffin-embedded tissue sections (n = 402) with histopathologically confirmed gastric adenocarcinoma were examined using immunohistochemistry. GATA3 was found to be localized in the nucleus, and the GATA3 immunoreactivity presented significant differences between the tumor tissue samples and the adjacent non-tumor tissue samples. Overall, for the tumor tissue samples, 225 cases (56%) displayed weak nuclear GATA3 expression and the remaining 177 cases (44%) showed positive or strongly positive GATA3 expression. Representative photomicrographs are shown in Figure 2.

### Association between GATA3 Expression and Clinicopathological Characteristics

By comparing the results of our RT-qPCR and immunohistochemical analyses of GATA3 expression with the clinicopathological characteristics of the patients, GATA3 expression was found to be significantly reduced in patients with large tumors (P = 0.017), signet ring cell carcinoma/mucinous carcinoma (P = 0.005), and tumors with lymphatic/venous invasion (P = 0.040). Decreased GATA3 expression was also observed significantly more frequently in tumors with deeper invasion (T4a and T4b) (P<0.001), tumors with a higher lymph node metastatic status (N2 and N3) (P<0.001) and cases with distant metastases (M) (P<0.001). Furthermore, 126 of the 205 (61.5%) patients with stage III disease and 39 of the 46 (84.8%) patients with stage IV disease had decreased GATA3 expression (P<0.001) (Table 1).

**Table 1:** Relationship between the GATA3 expression and clinicopathologic features of patients with gastric cancer (n = 402).

		GATA3 expression		
Variables	Number (%)	Low (n = 225)(56.0%)	High (n = 177)(44.0%)	*P value*
**Age(years)**				0.130
<60	226 (56.2%)	119 (52.7%)	107 (47.3%)	
≥60	176 (43.8%)	106 (60.2%)	70 (39.8%)	
**Gender**				0.181
Male	272 (67.7%)	146 (53.7%)	126 (46.3%)	
Female	130 (32.3%)	79 (60.8%)	51 (39.2%)	
**Tumor size (cm)**				0.017*
≤5.0	251 (62.4%)	129 (51.4%)	122 (48.6%)	
>5.0	151 (37.6%)	96 (63.6%)	55 (36.4%)	
**Histological grade**				0.005*
Well/Moderately differentiated (G1/2)	128 (31.8%)	55 (43.0%)	73 (57.0%)	
Poorly differentiated (G3)	227 (56.5%)	143 (63.0%)	84 (37.0%)	
signet ring cell carcinoma/Mucinous Carcinoma(G4)	47 (11.7%)	27 (57.4%)	20 (42.6%)	
**Location**				0.096
Proximal	224 (55.7%)	138 (61.6%)	86 (38.4%)	
Distant	156 (38.8%)	72 (46.2%)	84 (53.8%)	
Total	22 (5.5%)	15 (68.2%)	7 (31.8%)	
**Lymphatic/Venous Invasion**				0.040*
No	366 (91.0%)	199 (54.4%)	167 (45.6%)	
Yes	36 (9.0%)	26 (72.2%)	10 (27.8%)	
**Tumor invasion (T)**				<0.001*
T1	32 (8.0%)	9 (28.1%)	23 (71.9%)	
T2	38 (9.5%)	18 (47.4%)	20 (52.6%)	
T3	74 (18.4%)	41 (55.4%)	33 (44.6%)	
T4a	213 (53.0%)	127 (59.6%)	86 (40.4%)	
T4b	45 (11.1%)	30 (66.7%)	15 (33.3%)	
**Nodal status (N)**				<0.001*
N0	127 (31.6%)	55 (43.3%)	72 (56.7%)	
N1	78 (19.4%)	43 (55.1%)	35 (44.9%)	
N2	85 (21.1%)	49 (57.6%)	36 (42.4%)	
N3	112 (27.9%)	78 (69.6%)	34 (30.4%)	
**Metastasis status (M)**				<0.001*
M0	356 (88.6%)	186 (52.2%)	170 (47.8%)	
M1	46 (11.4%)	39 (84.8%)	7 (15.2%)	
**TNM Staging**				<0.001*
Stage I	51 (12.7%)	17 (33.3%)	34 (66.7%)	
Stage II	100 (24.9%)	43 (43.0%)	57 (57.0%)	
Stage III	205 (51.0%)	126 (61.5%)	79 (38.5%)	
Stage IV	46 (11.4%)	39 (84.8%)	7 (15.2%)	

* Statistically significant (*P*<0.05).

### Correlation between GATA3 Expression and Prognosis

**Figure 3:**
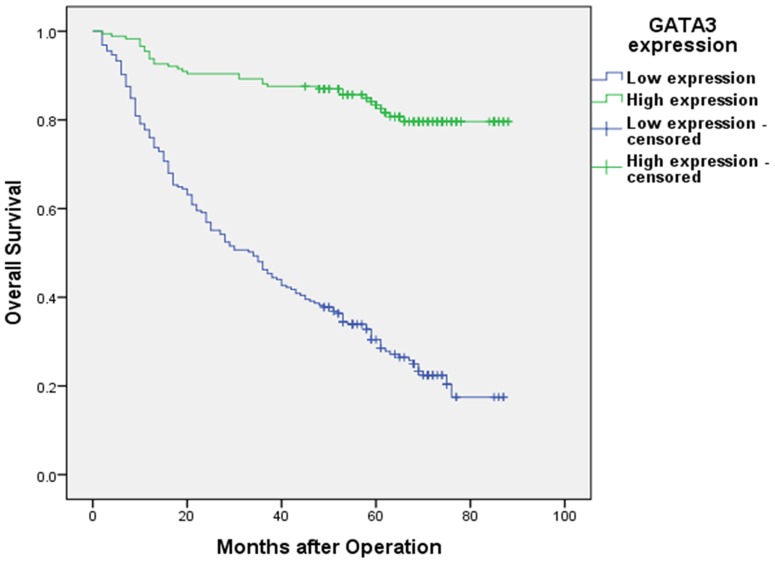
Kaplan-Meier survival curves of gastric cancer patients (n = 402) after gastrectomy. The survival rate of the patients in the GATA3-low group (n = 225) was significantly lower than that of the patients in the GATA3-high group (n = 177) (log-rank test, P<0.001).

The 1-, 3-, and 5-year overall survival rates in this cohort were 86.2%, 63.8%, and 52.4%, respectively. The 5-year overall survival rates in patients with high and low GATA3 expression were 83.4% and 30.5%, respectively. The overall survival of patients with low GATA3 expression was significantly shorter than that of patients with high GATA3 expression (P<0.001, log-rank test, Figure 3).

### Univariate and Multivariate Analysis

Univariate analysis showed that age (P = 0.041), tumor size (P<0.001), lymphatic/venous invasion (P<0.001), GATA3 expression (P<0.001), depth of tumor infiltration (P<0.001), lymph node metastasis (P<0.001), and distant metastasis (P<0.001) were significantly related to overall survival. Then, to compare the prognostic significance of GATA3 gene expression, two multivariate Cox regression analyzed models were constructed with or without containing the GATA3 gene expression. Model A (without containing GATA3 expression) confirmed age (P = 0.003), depth of tumor infiltration (P<0.001), lymph node metastasis (P<0.001), and distant metastasis (P<0.001) as independent predictors of the overall survival of patients with gastric adenocarcinoma. Model B (containing GATA3 expression) confirmed age (P = 0.006), GATA3 expression (P<0.001), depth of tumor infiltration (P<0.001), lymph node metastasis (P<0.001), and distant metastasis (P<0.001) as independent predictors of the overall survival of patients with gastric adenocarcinoma. The relative risk of death in patients with low GATA3 expression in their tumors was 5.375-fold higher than that of patients with high GATA3 expression in their tumors (HR = 5.375, 95% CI = 3.647–7.921) (Table 2).

**Table 2:** Univariate and multivariate survival analysis of clinic-pathologic variables in 402 cases of gastric carcinoma patients.

Model A: Model without containing the GATA3 expression						
	Univariate analysis			Multivariate analysis		
Variables	HR	95%CI	P value	HR	95%CI	P value
Gender (male vs. female)	1.163	0.869–1.558	0.310			
Age (year), (>60 vs. ≤60)	1.335	1.011–1.764	0.041*	1.534	1.155–2.039	0.003*
Location (distal/proximal/total)	0.783	0.608–1.009	0.059			
Size (cm) (>5 vs. ≤5)	1.723	1.303–2.277	<0.001*	1.157	0.865–1.548	0.325
Differentiation (G4/G3/G2&1)	1.202	0.972–1.487	0.090			
Lymphatic/Venous invasion (Yes vs. No)	2.374	1.568–3.593	<0.001*	1.526	0.995–2.339	0.053
T (T4b/T4a/T3/T2/T1)	1.906	1.602–2.267	<0.001*	1.572	1.311–1.886	<0.001*
N (N3/N2/N1/N0)	1.618	1.430–1.831	<0.001*	1.357	1.189–1.549	<0.001*
M (M1 vs. M0)	6.294	4.454–8.894	<0.001*	3.865	2.701–5.532	<0.001*

Model B: Model containing the GATA3 expression						
	Univariate analysis			Multivariate analysis		
Variables	HR	95%CI	P value	HR	95%CI	P value
Gender (male vs. female)	1.163	0.869–1.558	0.310			
Age (year), (>60 vs. ≤60)	1.335	1.011–1.764	0.041*	1.486	1.118–1.976	0.006*
Location (distal/proximal/total)	0.783	0.608–1.009	0.059			
Size (cm) (>5 vs. ≤5)	1.723	1.303–2.277	<0.001*	1.219	0.914–1.625	0.177
Differentiation (G4/G3/G2&1)	1.202	0.972–1.487	0.090			
Lymphatic/Venous invasion (Yes vs. No)	2.374	1.568–3.593	<0.001*	1.331	0.865–2.046	0.194
GATA3 (low vs. high)	6.580	4.495–9.634	<0.001*	5.375	3.647–7.921	<0.001*
T (T4b/T4a/T3/T2/T1)	1.906	1.602–2.267	<0.001*	1.662	1.380–2.001	<0.001*
N (N3/N2/N1/N0)	1.618	1.430–1.831	<0.001*	1.312	1.155–1.490	<0.001*
M (M1 vs. M0)	6.294	4.454–8.894	<0.001*	3.123	2.191–4.452	<0.001*

* Statistically significant (*P*<0.05).

Furthermore, to investigate the predictive ability of the aforementioned two models, Harrell's c-index was calculated as a measure of predictive accuracy of survival outcome. The c-index of 0.5 indicates accuracy similar to random guessing, and that of 1.0 indicates 100% predictive accuracy. In our study the c-index of Harrell were performed using R packages “rms”(http://cran.r-project.org/web/packages/rms/index.html) and the c-index values revealed that model B (c-index = 0.897) had superior discrimination ability to model A (c-index = 0.811).

## Discussion

Tumor progression arises as a consequence of a series of cellular events, which involve but are not limited to deregulation of cell proliferation, resistance to apoptosis, enhanced cell motility, augmented angiogenic potential, and anomalies in cell-cell interactions and the microenvironment, resulting in tumor formation, invasion and metastasis [29]. The process associated with tumor progression is precisely regulated by a small subset of genes that act by either enhancing (oncogenes) or diminishing (tumor suppressor genes) the final malignant outcome [30]–[34]. GATA3 is 1 of 6 members of the GATA zinc finger transcription factor family and plays an important role in promoting and directing cell proliferation, development, and differentiation in many tissues and cell types [9], [10]. GATA3 is a critical regulator of the development of various systems in both mice and humans [35]. The function of GATA3 has been extensively studied in T cell development and has recently been shown to be a downstream target of Notch in the Notch-mediated differentiation of TH2 cells [36], [37]. It is expressed in both hematopoietic and non-hematopoietic tissues, including the kidney, skin, mammary gland, and central nervous system [12], [22], [38]. Decreased expression of GATA3 in luminal breast cancer is associated with poor clinical outcome [11]. Therefore, the GATA3 expression level may be a promising prognostic biomarker [39].

Previously, statistics have shown a consistent downregulation of GATA3 in many tumor types, including breast, bladder, kidney, and ovary, when compared with normal tissues of the same anatomical origin [40]. Specifically, using tissue microarray and immunohistochemistry, it was found that GATA3 expression is inversely correlated with metastasis and overall survival in luminal cell carcinoma [28] However, to date, the prognostic significance of GATA3 in gastric adenocarcinoma has not been evaluated. In the current study, we estimated the expression of GATA3 in gastric adenocarcinoma by real-time qPCR and immunohistochemistry, in addition to analyzing its clinicopathological and prognostic significance in a relatively large number of human samples. We illustrated that GATA3 is expressed at lower levels with respect to both its mRNA and protein in gastric adenocarcinoma tissues compared with corresponding non-cancerous mucosa (P = 0.0014), in agreement with previous statistics shown in other types of tumor samples [23], [39]–[41].

For GATA3 to regulate gene expression, it must translocate from the cytoplasm into the nucleus to access its target genes. GATA3 contains a classical nuclear import signal and is transported into the nucleus by importin-α [42]. The binding of GATA3 to importin-α is regulated by phosphorylation, which is mediated by p38 mitogen-activated protein kinase (MAPK) and serves to enhance nuclear transport [43].

Likewise, in the current study, we observed an exclusively nuclear expression pattern of the GATA3 protein in gastric adenocarcinoma tissues. Previous studies have reported that other transcription factors exhibit both cytoplasmic and nuclear expression in many human malignancies [31], [44]. To explain this difference in localization, Wang et al. suggested that immunohistochemistry might only evaluate the end products of gene expression [31]. We speculated that some methodological factors, such as tissue processing, the heterogeneity of different types of malignancies and antigen specificity, may be contributing factors. In addition, modification of GATA3 or changes in the GATA3 protein itself may also interfere with these results. Our results showed significantly decreased expression of GATA3 in gastric cancer tissues and confirmed the expression of GATA3 as an independent risk factor for primary gastric adenocarcinoma patients.

In a mouse model of breast cancer, GATA3 was found to inhibit the metastatic seeding of breast cancer cells [45]. When overexpressed in a cell line selected for its high metastatic potential to the lung, GATA3 reduced tumor burden and metastasis. It was additionally noted that in this particular model, the ability of GATA3 to suppress metastasis was uncoupled from its ability to promote the differentiation of malignant mammary epithelial cells. This conclusion was reached based on the fact that GATA3 downregulated prometastatic genes and upregulated genes that inhibit metastasis but did not affect luminal differentiation markers [45]. These previous data support the assumption that GATA3 is not only a tumor suppressor gene in several types of human cancer but also plays a role in cancer differentiation.

Likewise, our observations are consistent with the idea that GATA3 acts as a tumor suppressor and suggest that it might play an important role in tumor progression in gastric cancer. In our study, which encompassed a relatively large number of gastric cancer patients (n = 402), low GATA3 expression was associated with tumors with deeper invasion (T) (P<0.001), tumors with a higher lymph node metastatic status (N) (P<0.001), cases with distant metastases (M) (P<0.001) and tumors with a later TNM stage (P<0.001). Consistent with our findings, decreased GATA3 expression was reported to be significantly associated with a higher grade of invasive bladder cancer [41]. Kaplan-Meier survival analysis showed a significant correlation between low GATA3 expression and poor clinical outcome in gastric cancer patients after operation. Cox hazard ratio regression analyses further demonstrated that the GATA3 expression level is an independent risk factor for overall survival, suggesting that this value may serve as a prognostic biomarker for gastric cancer patients after surgery. Furthermore, in our study the c-index values of Harrell using R packages revealed that the model containing GATA3 expression (c-index = 0.897) had superior discrimination ability to that without containing it (c-index = 0.811). These data suggest that examination of GATA3 expression might be helpful in guiding clinical management. However, the functional role and mechanisms of GATA3 in gastric cancer are unclear and require further investigation.

## Conclusions

In conclusion, the present study suggests that GATA3 expression is correlated with the clinicopathological parameters of gastric cancer patients and that its low expression independently predicts worse overall survival in patients with gastric adenocarcinoma. However, the molecular mechanisms involved in the regulation of GATA3 in gastric cancer require further investigation. Future studies in this field are necessary, as a better understanding of the function of GATA3 in malignancies has the potential to improve the prognosis of gastric cancer. Moreover, we expect that GATA3 may function as a useful target for new therapeutic interventions against gastric adenocarcinoma.

## Materials and Methods

### Ethics Statement

This research was approved by the Ethics Committee of the Sun Yat-sen University Cancer Center, and written informed consent was obtained from each patient involved in the study.

### Patients

Clinicopathological data from 402 gastric cancer patients who underwent surgical resection at Sun Yat-sen University Cancer Center from January 2003 to December 2006 were retrospectively analyzed. Patients who met the following eligibility criteria were included: (1) diagnosis of gastric adenocarcinoma identified by histopathological examination; (2) surgical history that included gastrectomy plus lymphadenectomy (limited or extended); (3) availability of complete follow-up data; (4) no preoperative treatment, such as chemotherapy or radiotherapy; (5) no history of familial malignancy or other synchronous malignancy (such as GIST, esophageal cancer, or colorectal cancer); (6) no recurrent gastric cancer or remnant gastric cancer; and (7) no death in the perioperative period. Tumor resection and D2 lymphadenectomy were performed by experienced surgeons, and the surgical procedures, which followed the Japanese Gastric Cancer Association (JGCA) guidelines [46], were similar for all patients who underwent radical resections.

Fresh gastric cancer and adjacent non-tumor tissue samples were obtained from 38 gastric cancer patients who underwent surgical resection at the Sun Yat-sen University Cancer Center between 2009 and 2011. After surgical resection, the fresh tissue samples were immediately immersed in RNAlater (Ambion, Inc., USA) and stored at 4°C overnight to allow for thorough penetration of the tissues; the samples were then frozen at −80°C until RNA extraction. Both the tumor tissue and the adjacent non-tumor tissue, which was located more than 2 cm away from the tumor tissue, were sampled and then verified by pathological examination. Paraffin-embedded samples were obtained from the 402 gastric cancer patients who underwent surgical resection at the Sun Yat-sen University Cancer Center between 2003 and 2006. Each tumor sample was assigned a histological grade based on the World Health Organization (WHO) classification criteria. All of the patients were staged using the 7th edition of the International Union Against Cancer (UICC) Tumor-Node-Metastasis (TNM) staging system [47].

### Extraction of Total RNA and Real-time Quantitative PCR

Total RNA was extracted using TRIzol solution (Invitrogen, USA) according to the manufacturer's protocol. RNAse-free DNAase I was used to eliminate any DNA contamination. The total RNA concentration and quantity were assessed by the absorbance at 260 nm using a NanoDrop spectrophotometer (ND-1000; Thermo Scientific, USA). Reverse transcription (RT) was performed using 2 µg of total RNA treated with M-MLV reverse transcriptase to synthesize first-strand cDNA (Promega, USA) according to the manufacturer's recommendations. The resulting cDNA was subjected to real-time quantitative PCR for the evaluation of the relative expression levels of GAPDH (as an internal control) and GATA3. The sequences of the sense and antisense primers were as follows: 5′-AGCCAGGAGAGCAGGGACG-3′(F) and 5′- CTGTTAATATTGTGAAGCTTGTAGTAGAG-3′(R) for GATA3; 5′- ATCACCATCTTCCAGGAGCGA-3′(F) and 5′-CCTTCTCCATGGTGGTGAAGAC-3′(R) for GAPDH. Gene-specific amplification was performed using an ABI 7900HT real-time PCR system (Applied Biosystems), which measured the binding of SYBR Green I to the double-stranded DNA. The reactions were performed in a total volume of 15 µl, which contained the following: 0.5 µl cDNA, 7.5 µl of 2×SYBR Green master mix (Invitrogen, USA), and 200 nM of each pair of oligonucleotide primers. The amplification was performed as follows: an initial step at 95°C for 10 min, followed by 45 cycles of 95°C for 30 sec and 60°C for 60 sec. The resolution curve was measured at 95°C for 15 sec, 60°C for 15 sec and 95°C for 15 sec. Regression curves were calculated for each sample, and the relative quantity of the amplified product was calculated from the threshold cycles using the instrument's software (SDS 2.3). The RT-qPCR amplicons were analyzed by gel electrophoresis to confirm the specificity of the generated products. The relative expression levels of the target genes were normalized to the geometric mean of the internal control gene GAPDH. Each sample was tested in duplicate, and each experiment was repeated at least twice using cDNA samples from separate reverse transcription reactions. The generated data were averaged and expressed in relative units of normalized expression. The data were analyzed using the comparative threshold cycle (2-ΔΔCT) method.

### Immunohistochemistry Analysis

The tissue sections were deparaffinized with dimethylbenzene and rehydrated with 100%, 95%, 90%, 80% and 70% ethanol. After three washes in phosphate-buffered saline (PBS), the slides were boiled in antigen retrieval buffer containing 0.01 M sodium citrate-hydrochloric acid (pH 6.0) for 15 min in a microwave oven. After rinsing with PBS, the tissue sections were incubated with primary antibody and then rinsed in 3% peroxidase quenching solution (Invitrogen) to block endogenous peroxidase. The sections were incubated with a rabbit monoclonal antibody against GATA3 (1∶200; Cell Signaling Technology, USA) at 4°C overnight and then with horseradish peroxidase (HRP) (ChemMate EnVision Detection Kit; Dako, Denmark) at room temperature for 30 min.

After washing with PBS, the signal was developed with a 3,3′-diaminobenzidine (DAB) solution, and all of the slides were counterstained with hematoxylin. As negative controls, adjacent sections were processed as described above, except that they were incubated overnight at 4°C in blocking solution without primary antibody.

The specimens were analyzed by three observers (R.P.K., W.W., and Y.Z.) who were blinded to the patients' clinical outcomes. Discrepancies between the observers were found in less than 10% of the examined slides, and a consensus was reached after further review. The total GATA3 immunostaining score was calculated as the sum of the percentage of positively stained tumor cells and the staining intensity and ranged from 0 to 9. Briefly, the percentage of positive staining was scored as 0 (0–9%, negative), 1 (10%–25%, sporadic), 2 (26%–50%, focal) or 3 (51%–100%, diffuse), and the intensity was scored as 0 (no staining), 1 (weak staining), 2 (moderate staining) or 3 (dark staining). The expression level of GATA3 was defined as follows: “−” (negative, score of 0), “+” (weakly positive, score of 1–3), “++” (positive, score of 4–6), “+++” (strongly positive, score of 7–9). We defined strong GATA3 expression as a total score of >3, and weak GATA3 expression as a total score of <3.

### Statistical Analysis

Differences in mRNA and protein expression between tumor samples and the paired adjacent non-tumor tissue samples were evaluated with the paired-samples t-test. The x^2^ test was used to analyze the relationships between GATA3 expression and various clinicopathological parameters. Survival curves were calculated using the Kaplan-Meier method and compared by the log-rank test. The Cox proportional hazard regression model was used for univariate and multivariate analyses to study the effects of the clinicopathological variables and GATA3 expression on survival. The statistical analyses were performed with the Statistical Package for the Social Sciences (version 17.0; SPSS Inc., Chicago, IL, USA), and a two-sided P value of <0.05 was considered to be statistically significant.
